# Intravenous Contrast is Associated with AKI in Patients with Stage 1–3 CKD: CON

**DOI:** 10.34067/KID.0000000000000187

**Published:** 2023-06-08

**Authors:** Jeffrey M. Turner

**Affiliations:** Section of Nephrology, Department of Internal Medicine, Yale School of Medicine, New Haven, Connecticut

**Keywords:** AKI and ICU nephrology, AKI, CKD, contrast, renalism

It is important to appreciate that contrast-induced AKI is an umbrella term that includes a number of different clinical scenarios. The first reports of this in the 1950s after exposure to hyperosmolar iodinated contrast during intravenous (IV) pyelography, or subsequent reports in the 1970s involving large-volume intra-arterial administration of contrast for angiographic procedures, are distinct clinical situations compared with the low-volume, iso-osmolar iodinated contrast that is routinely used for computed tomography (CT) imaging today.^1,2^ Appreciating this perspective is important to understanding the argument being put forth here, which is that AKI directly induced by IV administration, as opposed to intra-arterial administration, of contrast using contemporary administration volumes and formulations does not occur in patients with CKD stage 1–3. Another, and perhaps a more useful, way of framing this argument is that patients with less severe CKD can routinely get IV contrast imaging studies without the need for prophylaxis or worry.

Over the past decade or more, various authors have put forth provocative headlines in articles questioning whether contrast nephropathy is a real entity or just a myth.^3–5^ The purposeful use of these attention-grabbing headlines was in response to the pervasive overestimation of AKI risk from contemporary contrast agents that too often resulted in patients with CKD not getting beneficial tests or procedures, what has come to be known as renalism.^6^ Fear-mongering semantics aside, the opinion that contrast-induced AKI no longer exists was derived from studies that began being published around 2010, and that demonstrated that the risk of contrast-induced AKI from iso-osmolar contrast agents was drastically different compared with older studies in the literature that used large volumes, and often administered intra-arterial, of hyperosmolar contrast agents.

When considering the risk of AKI after IV contrast in patients with CKD stage 1–3, it is important to appreciate that there are no randomized control trials that address this question. It is this lack of data from randomized control trials which leaves room for controversy to exist because this puts limits on our ability to assess the true cause and effect of IV contrast and AKI. In addition, a major limitation to the currently available data are that there is significant selection bias in nonrandomized cohorts with moderate-to-advanced CKD because clinicians are typically more selective in who they give contrast to in this group. Another related point that needs to be considered is that within the population of patients undergoing contrast-enhanced CT scans, there are many alternative risk factors of AKI. This results in a high false-positive rate when defining contrast-induced AKI as a rise in serum creatinine in the subsequent days after contrast exposure. In an acknowledgment of this point, a recent consensus statement put out jointly by the American College of Radiology and the National Kidney Foundation promotes the use of the terms contrast-associated AKI and contrast-induced AKI to be used with a specific purpose.^7^ The former term acknowledging the lack of clarity in establishing cause and effect and the latter when clarity exists for cause and effect.

Despite these limitations, there is a growing body of evidence that we can reasonably rely on to draw consensus from. In these studies, the researchers have used advanced statistical methods to address the potential confounding pitfalls that exist among the observational cohorts they analyze. More specifically, they have used propensity score matching, which allows adjustment for confounders in nonrandomized cohorts and facilitates less biased comparisons between the study and control groups.^8^

In one of the larger cohorts involving 21,372 patient incidents that were compared based on 1:1 propensity score matching between those who got contrast-enhanced CT scans versus those who got unenhanced CT scans, no difference was found for risk of AKI.^4^ This lack of difference in risk persisted across the three groups stratified by baseline serum creatinine (<1.5 mg/dl; 1.5–2.0 mg/dl; or >2.0 mg/dl). Similar findings were reported by Hinson *et al.* in a separate study.^9^ Risk of AKI was the same in the patients in propensity-matched groups who received contrast versus those who did not, and this persisted across subgroups defined by different baseline eGFRs (15–29, 30–44, 45–59, 60–89, and ≥90). In addition, the findings in this study were unchanged when two separate definitions for AKI were interchanged (Acute Kidney Injury Network/Kidney Disease Improving Global Outcomes: rise in creatinine 0.3 mg/dl or contrast induced nephropathy criteria: rise in creatinine of 0.5 mg/dl). Several other large cohort studies using similar methods have also reported no increased risk of AKI from IV contrast in patients with eGFRs >30.^10–12^ Table 1 summarizes the findings from these various studies.

**Table 1.** Summary of findings in participants with GFR >30 from propensity-matched studies for AKI risk after intravenous contrast exposure

Study	Year	No. of Participants in Propensity Score–Matched Cohorts with eGFR >30	Findings
Davenport *et al.*^11^	2013	17,536	Overall no increased risk of AKI in those who received IV contrast and had an eGFR >30 In the subset of participants with an eGFR of 30–44, the OR for AKI was not statistically significant. (OR, 1.40; 95% CI, 0.997 to 1.97). This group included 1089 participants of the total 17,536 included in the PS-matched cohort with an eGFR >30
McDonald *et al.*^4^	2013	19,456^a^	In participants with a serum creatinine of ≤2.0 mg/dl, there was no increased risk of AKI from IV contrast. In addition, there was also no increased risk in the 1916 participants with a serum creatinine >2.0 mg/dl
Hinson *et al.*^9^	2017	8324^b^	No increased risk of AKI for participants with an eGFR of 30–89 (OR, 1.01; 95% CI, 0.96 to 1.06 for eGFR 30–44) (OR, 1.02; 95% CI, 0.97 to 1.06 for eGFR 45–59) (OR, 1.00; 95% CI, 0.98 to 1.02 for eGFR 60–89)
Tao *et al.*^12^	2018	420^c^	No increased risk of AKI from IV contrast in those with an eGFR or 30–89. In those with an eGFR of 60–89, patients not exposed to contrast had a higher risk of AKI. (OR, 0.65; 95% CI, 0.26 to 1.63 for eGFR 30–59) (OR, 0.27; 95% CI, 0.10 to 0.75 for eGFR 60–89)
Kene *et al.*^13^	2021	10,806	There was an increased risk of AKI in those receiving IV contrast with CKD stage 3. (ARR, 1.61; 95% CI, 1.42 to 1.80). However, this risk was not seen in the 372 participants with CKD stage 4–5 analyzed in the study (ARR, 1.41; 95% CI, 0.96 to 2.08)

IV, intravenous; OR, odds ratio; CI, confidence interval; mg/dl, milligram per deciliter; ARR, adjusted risk ratio.

a Participants in this study were grouped by serum creatinine, not eGFR. The reported number of participants in this table includes only those with a serum creatinine of <2.0 mg/dl. It excludes those participants with a serum creatinine of >2.0 mg/dl.

b This is the total number of participants with an eGFR of 30–89 in this study before propensity score (PS) matching. It was not reported how many participants were included in the analysis after PS matching occurred.

c Total number of participants with an eGFR of 30–89 after PS matching occurred.

In addition to serum creatinine–based AKI outcomes, multiple studies have evaluated the risk of more meaningful clinical outcomes, including emergent dialysis and short-term mortality, and no difference was seen in any subgroups irrespective of their baseline eGFR.^13–15^

Despite these data, some opinions have still been put forth suggesting an increased risk in patients with a GFR of 30–45. Typically, these opinions cite a single propensity score matching study that stratified participants by eGFR groupings.^11^ The findings of this study did in fact demonstrate an increased risk in those with a baseline eGFR of <30 as compared with those with an eGFR ≥60 (odds ratio, 2.97; *P* = 0.02). However, for those with an eGFR of 30–44, there was no statistically significant higher risk of AKI when compared with those with an eGFR of ≥60 (odds ratio, 1.40; 95% confidence interval, 0.997 to 1.97). In another study by Kene *et al.*, a 4.8% higher risk of contrast-induced AKI was found in patients with CKD stage 3. This was in a population being cared for in the emergency department, but surprisingly, this higher risk was neither found in those with more severe CKD stages 4–5, nor did it reflect a higher rate of dialysis at 30 days in any group.^13^ Whether the study design or population in this analysis were the reason for the contrasting results is not clear, but when evaluated in the context of the other data, it is difficult to find useful conclusions from this study alone to extrapolate to other populations.

There is little debate that the risk of contrast-induced AKI has evolved after the routine practice of using iso-osmolar agents in recent decades. With that being said, much debate continues regarding what populations still have clinically meaningful risks that warrant special attention. Although the data are limited, clinicians should reasonably accept the conclusion based on the available body of evidence that patients with CKD stage 1–3 should not be considered in this group. Barring concerns of possible superimposed AKI at the time of their imaging study, these patients should not be subjected to routine therapies for prophylaxis or avoidance of medically indicated studies because of fears about IV contrast administration. Guidelines and institutional polices should reflect this and current practice standards should incorporate it.

## Disclosures

J.M. Turner reports the following—consultancy: BOA Biomedical and Boehringer Ingelheim.
